# Unravelling networks in local public health policymaking in three European countries – a systems analysis

**DOI:** 10.1186/s12961-016-0168-2

**Published:** 2017-02-03

**Authors:** Hilde P. E. M. Spitters, Cathrine J. Lau, Petru Sandu, Marcel Quanjel, Diana Dulf, Charlotte Glümer, Hans A. M. van Oers, Ien A. M. van de Goor

**Affiliations:** 1 Department of Tranzo, Tilburg School of Social and Behavioral Sciences, Tilburg University, P.O. Box 90153, 5000 LE, Tilburg, The Netherlands; 2 Research Centre for Prevention and Health, Capital Region of Denmark, Ndr, Ringvej 57, Afsnit 84/85, 2600 Glostrup, Denmark; 3 Department of Public Health, College of Political, Administrative and Communication Sciences, Babes-Bolyai University, 7 Pandurilor St. Universitas, Room 910, 400376 Cluj-Napoca, Romania; 4 Youth Care, Special Needs Education and Research, P.O. Box 6546, 6503 GA, Nijmegen, The Netherlands; 5 Department of Health Sciences and Technology, Aalborg University, Frederik Bayers vej 7D2, DK-9220 Aalborg, Denmark; 6 National Institute of Public Health and the Environment (RIVM), P.O. Box 1, 3720 BA Bilthoven, The Netherlands

**Keywords:** Schematic model, Systems analysis, Stakeholder network, Local policymaking process, Relations, Public health

## Abstract

**Background:**

Facilitating and enhancing interaction between stakeholders involved in the policymaking process to stimulate collaboration and use of evidence, is important to foster the development of effective Health Enhancing Physical Activity (HEPA) policies. Performing an analysis of real-world policymaking processes will help reveal the complexity of a network of stakeholders. Therefore, the main objectives were to unravel the stakeholder network in the policy process by conducting three systems analyses, and to increase insight into the similarities and differences in the policy processes of these European country cases.

**Methods:**

A systems analysis of the local HEPA policymaking process was performed in three European countries involved in the ‘REsearch into POlicy to enhance Physical Activity’ (REPOPA) project, resulting in three schematic models showing the main stakeholders and their relationships. The models were used to compare the systems, focusing on implications with respect to collaboration and use of evidence in local HEPA policymaking. Policy documents and relevant webpages were examined and main stakeholders were interviewed.

**Results:**

The systems analysis in each country identified the main stakeholders involved and their position and relations in the policymaking process. The Netherlands and Denmark were the most similar and both differed most from Romania, especially at the level of accountability of the local public authorities for local HEPA policymaking. The categories of driving forces underlying the relations between stakeholders were formal relations, informal interaction and knowledge exchange.

**Conclusions:**

A systems analysis providing detailed descriptions of positions and relations in the stakeholder network in local level HEPA policymaking is rather unique in this area. The analyses are useful when a need arises for increased interaction, collaboration and use of knowledge between stakeholders in the local HEPA network, as they provide an overview of the stakeholders involved and their mutual relations. This information can be an important starting point to enhance the uptake of evidence and build more effective public health policies.

## Background

Public health policies aim to solve complex problems that involve many different parties and sectors. These problems are complex because they are influenced by many determinants inside and outside the health sector, including environmental and cultural factors [1, 2]. Therefore, in order to tackle these problems, working towards integrated public health policies has been advocated [3, 4]. Such integrated public health policies (also called cross-sectoral approaches) are necessary to enhance effective public health policymaking, requiring involvement of many stakeholders [5]. Furthermore, inspired by evidence-based medicine, the effectiveness of public health policies might be increased by integrating the best available evidence, i.e. research evidence, the evidence/expertise of stakeholders, as well as other types of evidence [6, 7].

Due to differences between the stakeholders’ backgrounds, points of view and expertise, facilitating and enhancing interaction between stakeholders involved in the policymaking process is essential [8, 9]. In a review on barriers and facilitators of the use of evidence by policymakers, Oliver et al. [10] highlighted the importance of understanding relations and collaboration between stakeholders. Stakeholders perceive relations as one of the main elements for the uptake of evidence in the policy process. Hence, the interaction and relationships stakeholders maintain with each other in a network (i.e. collaboration processes in policymaking), might play an important role in explaining collaboration [6, 7, 11–13] and, subsequently, in the exchange and uptake of evidence in policy processes [11, 13–18]. This is in line with the interaction model, which describes the utilisation process of knowledge in a stakeholder network. In this model, the interaction between researchers and other stakeholders in the network is highlighted, exposing them to each other’s worlds and organisations’ interests [19–23].

Local public health policies should be developed in accordance with national policies [24]. A priority area within public health policy is aiming at Health Enhancing Physical Activity (HEPA) [25], because of the high prevalence of overweight and obesity, and low rates of physical activity in most western societies. HEPA policymaking is a good example of the necessity of cross-sectoral collaboration to address issues such as overweight and physical activity. HEPA is highly relevant at local level, because of the many involved stakeholders to implement the policy locally [26–28]. Therefore, there is a need to get insight into the current local HEPA policymaking process.

To some extent, it is already known which local stakeholders (e.g. local government, policy advisors, researchers, local knowledge institutes) are involved in the local public health policymaking process, and what their relations are [11, 15, 28–32]. However, limited details are available on the relations between stakeholders in the network when looking at this local policymaking process as a whole. Therefore, a study exploring the relational network in the local public health policymaking process aiming at HEPA can help elucidate the mechanisms that influence the nature and extent of interaction and collaboration among stakeholders [24]. In this study, the term stakeholders refers to organisations, groups of persons or individuals who are influencing or are influenced by choices and regulations by another organisation [33]. Cross-sectoral collaboration involves partnerships between different sectors within the government, and between government, non-profits, private parties and the communities, and/or the public as a whole [34]. Private parties are enterprises with their own aims and interests and without direct financial support from the government.

One way to unravel the interactions within a stakeholder network and the processes at stake is to perform a systems analysis. A systems analysis focuses on the entire system and analyses interactions and relations between organisations in the stakeholder network, with the aim to unravel the relations within the network. In such an analysis, influencing elements, such as stakeholders and relations, are identified and visualised in a schematic representation [35–37]. The method reveals two major aspects of the policy network in the policy process – the structure of the network and its main stakeholders involved, and the relations (such as interaction, exchange and influence) between them [38]. The relations between the stakeholders are mainly characterised by driving forces; these can be seen as the representation of incentives underlying the relations that shape the policy process, in any given context.

The aim of this study was to analyse and compare the stakeholder networks in local HEPA policymaking in three European country cases in the Netherlands, Denmark and Romania. The main objectives were to unravel the stakeholder network in the policy process by conducting three systems analyses, and to increase insight into the similarities and differences in the policy processes of these European country cases.

## Methods

### Design

This study was performed within the framework of the FP7 project ‘REsearch into POlicy to enhance Physical Activity’ (REPOPA) [39]; this project conducted research in six European countries with the aim to understand and support the development of more evidence-informed policies in enhancing physical activity [39]. In REPOPA, the HEPA policies were used as an example to gain insight into cross-sector collaboration and the incorporation of evidence in the public health policymaking process. Three of the REPOPA countries, the Netherlands, Denmark and Romania, conducted a systems analysis [35–37] to reveal the complex cross-sectoral interactions that take place in a stakeholder network in a local policy process. This study mainly focused on the involvement of stakeholders in the policy process and on their mutual relations after the specific policy was approved and the implementation plan was to be formed, while keeping in mind the non-linear process of policy development.

### Inclusion criteria for the three cases

In each of the countries, a case was selected by the country team. The first inclusion criterion was that the case focused on the process of local HEPA policymaking. In this study, local level refers to the governmental authorities accountable for local HEPA policy. Depending on each country, the focus was more on local/municipal or regional/county level concerning a specific geographical area with several municipalities. As second criterion, the stakeholders of the case had to feel a need to explore the policymaking process in a more detailed way and enhance cross-sector collaboration. The third criterion was that stakeholders of the cases had to be willing to participate in the intensive process that is inherent in performing a systems analysis. See Table 1 for more information on context in terms of the national political structure and specific information of the three country cases.

**Table 1:** Context of the three country cases

	The Netherlands	Denmark	Romania
National political and administrative structure			
Political system	Parliament Democracy; representatives in the Parliament are chosen every 4 years	Decentralised administrative system	The executive power is represented by the Government, led by a Prime Minister, designated by the President of the state
Administrative structure	National authorities develop and present national planning policies and provide guidance for regional (regions) and local (municipal) level Decentralised administrative system with part of the tasks transferred from national to the local level, which affects health, and HEPA policies on local (municipal) level, ‘make the healthy choice the easy choice’	National level facilitates guidance and policies for regional and local level The political and administrative structures regarding the tasks given to the municipalities after the structural reform in 2007 vary significantly among the municipalities; there are three different overall models for organisation of health-related tasks – in some municipalities the new tasks are handled in a separate health department, other municipalities have chosen a model where the new tasks are incorporated in a larger department, for instance, together with social care; this is the most widespread model among the Danish municipalities. Finally, some municipalities have placed all health-related tasks in the central administration of the municipality	The National Government develop policies that are implemented at county and local level by the ministries representatives and by the public administrative authorities Although efforts are made for decentralisation the administrative system is still centralised, with minimal autonomy for county and local level structures in regards for policies development
Responsibility HEPA	Regions are accountable for planning, healthcare and recreation; municipalities are accountable for education, planning,welfare and social affairs	Regional level being responsible for healthcare services and local (municipal) level for health promotion The policy state, that the responsibility for the implementation of the policy is a common responsibility, which go across each of the sectors in the municipality; the policy does not mention concrete implementation initiatives, but state that such initiatives will be announced yearly	According to the law, the Ministry of National Education is the Governmental structure responsible for organising the physical education and sport activities in the pre-university and university system The ministry of Youth and Sport and its structures (i.e. national sport federations, ounty youth and sport directions) are responsible for organising the performance sport and the sport for all at national level The Ministry of Health (through the National Institute of Public Health) has a role in evaluating the health status and health-related behaviour of school aged population – including PA levels
Who develops the local public health policy, including HEPA	The policymaking process follows a 4-year prevention cycle, based on the Public Health Act. For local level, this means that every municipality, which is an autonomous authority with an elected city council, writes their own local policy document based on the national policy document (main priorities and recommendations) and on the epidemiologic information collected by the Community Health Services of the local health situation	Municipalities are autonomous authorities with elected councils The Parliament and the government are responsible for passing laws and developing general policies The Ministry of Health is the principal health authority, which presents specific action plans on how to implement the Parliament’s health policies	The county representatives of the national governmental structures have the role of administrating and implementing the governmental programs in the fields of HEPA and physical education and sport (i.e. the County Youth and Sport Department) and education (i.e. the County School Inspectorate, Children Palace, School Sports Club) Local and county public administrative authorities also assume some marginal responsibilities in maintaining and promoting population health, but very few (if none) responsibilities related to HEPA promotion; private and civil society representatives are also involved in HEPA promotion, not as part of any structured policy, but by implementing targeted programs (e.g. running, cycling, etc.)
Cases			
Size of the city:	Average size municipality	Average size municipality	Highly populated municipality with a high student population
Setting:	Local	Local	Local/county
Stage policy plan:	New developed health policy, working towards an implementation plan	New developed health policy and needed an implementation plan	HEPA policy plan was in the development phase
Focus policy plan	Focus on the Dutch national umbrella policy ‘Youth on Healthy Weight’ (in Dutch: JOGG) and the national health policy ‘Health close to the people (2011)’, with main focus youth and physical activity	Focus of the plan was on physical activity promotion, with the target group children and young adults and citizens with special needs and chronic diseases	One of the working groups on physical activity was specifically at local level and focusing on the development of the local HEPA Strategy for 2014–2020, ‘Sport and Community’
Theme	Specific HEPA policy	General health policy, including HEPA	Sports policy, including HEPA
Responsible local public health policy	The administrative level of the municipality was accountable for the HEPA policy plan; the responsibility of implementation of JOGG was assigned to the Regional Sport Service	Primarily the administrative level in the sector Health and Care was responsible for health promotion, but responsibility of the implementation of the policy is a common responsibility, which goes across each of the sectors in the municipality	None of the stakeholders is specifically responsible for the implementation of the policy plan

### Starting point for the systems analysis

For the systems analysis, an in-depth analysis of the local HEPA policymaking process and the policy network was conducted in the three selected country cases. Each country focused on one specific case (municipality or county). Local, regional and national level stakeholders were taken into account when these stakeholders’ relations had a direct influence on the local HEPA policymaking process or when these stakeholders had a specifically assigned role when the implementation plan was developed at local level.

The actual systems analysis took place separately in each country, and the results of the analyses were presented in a schematic model by the research team in each country. A Dutch expert in developing systems analyses facilitated the process in all three countries. The individual research teams discussed the development of their systems analysis by means of periodic conference calls. On two occasions, face-to-face meetings were held to validate the three systems analyses, with regard to schematic appearance and understanding of each other’s systems.

### Performing the systems analysis

A systems analysis is built on multiple data sources, ranging from written documents (i.e. policy documents, governmental websites) that provide a starting point, to interviews with key figures and stakeholders [9, 38]. Table 2 shows a summary of the sources of data collection for each of the three country cases.

**Table 2:** Data collection for the three country cases

The Netherlands	
Previous work REPOPA (Oct 2011–Jan 2013)	Data from interviews (14) with local, regional and national stakeholders on use of evidence in the process of developing 1 national and 1 local HEPA policy
Preparatory meetings with research team: focusing on context and specifics of the local setting with respect to HEPA policymaking	Research team: • Two researchers in Public Health Tilburg University • Two policy advisors (Dutch Institute for Healthcare Improvement) • One expert in conducting systems analyses
Previous research on cross sectoral policymaking, stakeholders and networks at local level in the Netherlands	- Aarts MJ. Children, physical activity and the environment [57] - De Goede J. Knowledge in process [19] - Hoeijmakers M. Local health policy development processes [58] - Van Egmond S. Science and policy in interaction [59]
Policy documents related to HEPA policy at national and local level^a^	- National level policy documents: six documents - Regional level policy documents: six documents - Local level policy documents of other municipalities: 14 documents - Local level policy documents of case: 12 documents
Semi-structured interviews (individual and group)	General level: Individual (three documents)^b^ and group (one document)^b^, role and institute:
	- Researcher on local public health policy, Tilburg University
	- Policy advisor, National Institute of Public Health and the Environment
	- Policy advisor, Regional Public Health Service West-Brabant
	- Two policy advisors, Regional Public Health Service Hart voor Brabant
	Case level: Individual (one document)^b^ and group (four documents)^b^, role and institute:
	- Two policy advisors, Regional Public Health Service West-Brabant (one time)
	- Policy advisor, Regional Sport Service West-Brabant
	- Key figure group case (three times): • One policy advisor, Regional Public Health Service West-Brabant • One policy advisor, Regional Sport Service West-Brabant • One policymaker, Municipality Dutch case
Denmark	
Previous work REPOPA (Oct 2011–Jan 2013)	Data from interviews (17) with local and regional stakeholders on use of evidence in the process of developing one regional and three local HEPA policies
Preparatory meetings for research team: focusing on context and specifics of the local setting with respect to HEPA policymaking	Research team • Two researchers/policy advisors of Research Centre for Prevention and Health • Two researchers in Public Health of University Southern Denmark
Books on cross sectoral policymaking, stakeholders and networks in Denmark	- Fischer-Nielsen B. Kommunalpolitik [60] - Lundtorp S, Rasmussen M. Rigtigt kommunalt – ledelse I kommuner og amter fra reform til reform [61]
Policy documents related to governance and HEPA policy at national and local level^a^	- International policy documents: one document - National level policy documents: 12 documents - National level law document: one document - Regional level policy documents: two documents - Local level policy documents from other municipalities: four documents - Local level policy documents of case: 10 documents
Discussion over email	General level: Individual (one document), role and institute:
	- Researcher/policy advisor, Local government Denmark (email contact)
Semi-structured interviews (individual and group)	Case level: Group (five documents)^b^, role and institute:
	- Key figure group case (four times face-to-face and once by telephone) • Three policymakers of Centre of Health, Sport and Citizenship (two from health and one from sports)
Romania	
Previous work REPOPA (Oct 2011–Jan 2013)	Data from interviews (four) with local, regional and national stakeholders on use of evidence in the process of developing two national HEPA policies
Preparatory meetings with research team: focusing on context and specifics of the local setting with respect to HEPA policymaking	Research team: • Three researchers in public health, Babes-Bolyai University
Policy documents related to HEPA policy at national and local level^a^	- International policy documents: four documents - National level policy documents: one document - Documentation from the actual local strategy of the case
Semi-structured interviews (individual)	National level: Individual (three documents)^b^, role and institute:
	- General Secretary of the National Sport for All Federation;
	- General Inspector, Ministry of Education;
	- Policy advisor, National Focal Point - HEPA Europe Network, National Institute of Public Health)
	Case level: Individual (27 documents)^b^, role and institute:
	*Local level public sector*
	- Three stakeholders city hall, two policy advisors and one director
	- Three stakeholders city council, two policy advisors and one director
	*County level public sector*
	- One stakeholder county council, director
	- Five stakeholders sector education, one inspector education, three directors, one assistant director (five different organisations)
	- Two stakeholders sector public health, one policy advisor, one director (two different organisations)
	- Four stakeholders sector sports, one dean, one director, one manager (three different organisations)
	*Local organisations*
	- Four stakeholders private sector, three directors, one press officer (four different organisations)
	- Five stakeholders civil society, five directors (four different organisations)
Websites for general information	Looked for documents on the official websites of public institutions at national and local level to explore multiple documents for each of these institutions

^a^Policy documents include national policies and local policies and implementation plans in public health, HEPA, Sports, policy evaluations, vision of the Aldermen and organisation diagrams, available on websites of local governance and national organisations

^b^The number in brackets refers to the number of conducted interviews

Based on Peters et al. [36], a guideline of four steps was developed and used by each team to carry out the systems analysis; as recommended, each country adapted the steps to their own specific context [35]; the four steps are described below.

The stepwise process was iterative, moving back and forth between document analysis and interviewing involved stakeholders; this was a qualitative and interpretative process. For a good understanding of the country stakeholder network, initially also policy documents of other municipalities were taken into account, before going into detail in the country case. The systems analysis took place during a 6-month period (April 2013 to September 2013).

The first step was to identify the stakeholder network in the real-life system, by exploring several policy documents, governmental websites and conducting interviews with key figures, see Table 2 for an overview of the data collected. The interviews were undertaken to identify stakeholders in the local HEPA policy process, as well as the problems and needs they experienced in local HEPA policymaking. This collaborative approach with key figures was used to acquire an overview of the stakeholder network and incorporate the stakeholders’ expertise early on in the design process [9, 38]. In the Netherlands and Denmark, after analysing the policy documents, the main stakeholders were identified relatively early in the process. To identify the main Romanian stakeholders involved in the local HEPA policymaking process, a snowball method was used; this started with identifying main local stakeholders to acquire a broader picture of whom to contact next [40]. This specific approach was used for Romania because analysis of the policy documents failed to reveal how and which stakeholders were involved in the process, at what point in time, in what way, and at what level. Interviewing the known stakeholders was essential to elucidate Romanian HEPA policymaking and identify main stakeholders at the relevant levels. Data triangulation was used for completeness [41, 42]. Depending on the country, interviews (including consultations with experts in the field) and policy documents (e.g. national and local health policies and strategies of different stakeholder institutes) were analysed, see Table 2 for an overview of the data collected.

The second step was mapping the relative position of the identified stakeholders in the stakeholder network, thereby creating the preliminary schematic model of the systems analysis. Because of the qualitative nature of the method, we have not measured exact distance, but interpreted the distance of relations by interviews and the verification step (step 4).

In this mapping phase, the positions of stakeholders towards each other in the HEPA policymaking process were taken into consideration. Stakeholders were placed in the preliminary schematic model based on the centrality of their role in the HEPA policymaking process and the level (local, regional/county or national) they acted on [28, 31, 41, 42]. Key figures from the local authorities and the regional health service provided information on this aspect. At this point, the relations between stakeholders were not yet analysed.

In the third step, the research team made an inventory and description of the type of relations between the identified stakeholders. Subsequently, these relations were analysed, interpreted and categorised by underlying driving forces, the main incentives for organisations to participate in the stakeholder network. Examples of such main incentives are advocacy, regulations and law or financial resources. The inventory of relations and the categorisation of driving forces was based on the input from the interviews, document analysis and discussion in the research team.

The relations were added to the preliminary schematic model of the systems analysis in step two. The types of relations are presented in the schematic models by arrows of different types, different colours, and in one- or two-way directions. Relations are included when impacting local HEPA policymaking, including relations relevant for the development of and the implementation of the HEPA policy plan.

In the fourth step, the schematic model of step three was verified. In all three countries, the schematic model of the systems analysis was verified in a dialogue between the country research teams and various key figures and experts such as policymakers, policy advisors, researchers, and other stakeholders involved in the local policymaking process. The country research team discussed the schematic model with some of the main stakeholders, which differed per country depending on availability. In the Netherlands, the schematic model was verified with two local policy advisors of a Regional Public Health Service with expertise in the local stakeholder network. In Denmark, the verification step was undertaken with the key person from the local authority and with researchers from Southern Denmark University with expertise in evidence-informed policymaking, who were also involved in steps 1 and 3. In Romania, different stakeholders from the county and local policy network were asked individually to verify the schematic model of the systems analysis and offer feedback. To finalise the analyses, adjustments were made accordingly. During this verification, focus was on the presence of all identified stakeholders in the stakeholder network, their roles and their mutual relations.

### Comparison between countries

Comparison between the three countries was undertaken in two steps, focusing on the similarities and differences between the three cases. First, comparison focused on the main stakeholders present in each network, at different levels. Second, the relations between stakeholders were compared, categorised by the three driving forces as determined in step 3. In this comparison, the focus was on implications for local HEPA policymaking.

## Results

### Description of the systems analyses

**Fig. 1:**
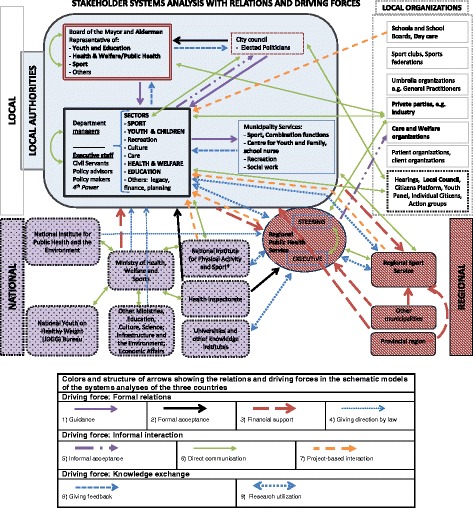
Schematic model of the systems analysis in the Netherlands. Legend of arrows showing the relations in the schematic models

**Fig. 2:**
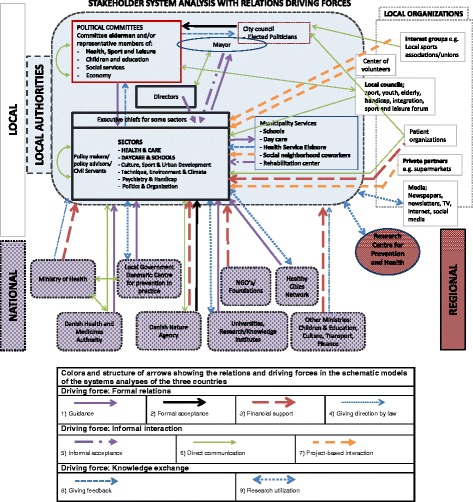
Schematic model of the systems analysis in Denmark. Legend of arrows showing the relations in the schematic models

**Fig. 3:**
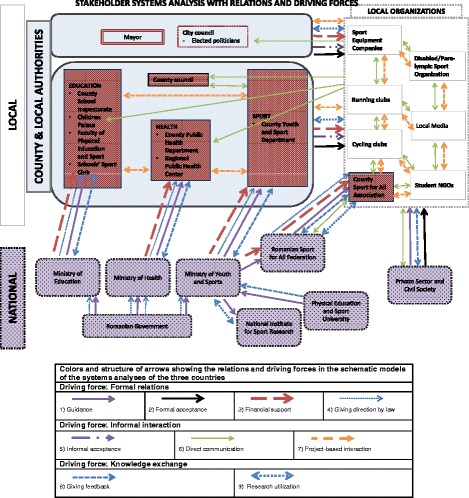
Schematic model of the systems analysis in Romania. Legend of arrows showing the relations in the schematic models

The systems analyses of the Dutch, Danish and Romanian cases are presented in Figs. 1, 2 and 3, respectively. The figures show the network in local HEPA policymaking in terms of stakeholders and their relations that influence the process.

The results below follow the four steps (described above) and are presented in the following order – the Netherlands, Denmark and Romania. Then, a comparison of the stakeholder networks is made, as shown in the three schematic models of the systems analyses.

### Step 1 – Main stakeholders

#### Dutch case

In the Netherlands, three levels that influence the development of the local HEPA policy in the stakeholder network were identified, the local, regional and national level (Fig. 1). Central in the local HEPA policymaking process were, at the local level, the health sector within the local authority or municipal government (see grey box in Fig. 1). At regional level key stakeholders were the Regional Public Health Service and the Regional Sport Service. Both these services work with or for several municipalities in the region and especially the Regional Public Health Service has a close relation with the local authorities.

The local authority consisted of several stakeholders with different roles in the HEPA policymaking process. The local authority stakeholders identified were the city council, the Board of Mayor and Aldermen, the different policy sectors (with civil officers) in the municipality, and specific municipality services (e.g. the centre for youth and family, and the sport service). Furthermore, within the municipality, other local organisations (apart from the local authorities) were identified in the stakeholder network; they play an important role in the policy process, as they work for or with the target groups of the local HEPA policy (white box in Fig. 1). Some secondary schools and care and welfare organisations work at both local and regional level; however, to avoid complexity, these are not shown in the Dutch schematic model.

Influencing stakeholders at national level include ministries (especially the Ministry of Health, Welfare and Sport) and national knowledge institutes, e.g. the National Institute for Public Health and the Environment, and the universities.

#### Danish case

In Denmark, three levels in the policy network that influence local HEPA policymaking were also identified, the local, regional and national level (Fig. 2). Central in this policymaking process is the health sector within the local authorities. The directors of the sectors and the Mayor comprise the management of the municipality and prioritise the initiatives across the municipality sectors, and therefore have a key role in local HEPA policymaking.

At the local level, the local authorities were the accountable entity (see grey box in Fig. 2). Other stakeholders in the local authorities were the city council, the political committees (e.g. health, sport and leisure), the sectors, and the municipality services (e.g. schools, and day care). Other stakeholders in the Danish local stakeholder network outside the local authorities were local organisations such as interest groups (e.g. local sport associations), private parties, patient organisations, volunteer centre, local councils of different citizen groups, and the media.

An influencing stakeholder in the local HEPA policy process at regional level was the knowledge institute Research Centre for Prevention and Health. At the national level, ministries (especially the Ministry of Health), other authorities (e.g. National Center for Local Governments Denmark, and the Danish Health and Medicines Authority) and various knowledge institutes were identified as having an influence on local HEPA policymaking.

#### Romanian case

In Romania, the systems analysis resulted in a schematic model showing a different picture of the stakeholder network. The main stakeholders involved in local HEPA policymaking were organised differently than those in the Dutch and Danish situation. In Romania, three levels were also identified, namely local, county (to some extent comparable with regional level), and national (Fig. 3). No central role was given to any of the identified stakeholders in regards to local HEPA policymaking, but the national level sport sector was to a greater extent responsible for HEPA policymaking in general. All local and county stakeholders were in charge of locally embedding this policy. Furthermore, many of the county public authority and local organisation stakeholders were representing their national level stakeholders.

At the local level, several main stakeholders with a role in local HEPA policymaking were identified, i.e. the Mayor’s office, and the city council. They are held responsible for the health status and overall wellbeing of the population; these roles derive from the responsibilities for health promotion, including physical activity. Other local stakeholders are the local organisations (including private companies and NGOs), that support the public strategies and conduct their own programs and events.

At the county level, the departments are in charge of implementation of strategies developed at the national level. In the sports sectors, the County Youth and Sport Department is the main stakeholder in charge of implementing the strategies developed at national level. This stakeholder worked together with the county Sport for All Association, and other locally-embedded public (e.g. county council, school inspectorate, public health department) and local organisations (e.g. running clubs, sport equipment companies, students’ NGOs), in the implementation of sport programs and events. The role of these county level stakeholders in actual local HEPA policymaking is very limited, as their accountability and expertise focuses on implementation of the nationally developed strategies. In addition, the county Sport for All Association is considered an NGO, even though it falls under the national Sport for All Federation, within the Ministry of Youth and Sport.

At the national level, the Ministry of Youth and Sport is the main stakeholder in charge of developing the Sport for All Strategy. The Romanian Sport for All Federation is the stakeholder appointed by this Ministry to work on this strategy, and is seen as the liaison between the sports sector and the county Sport for All Association. Other national stakeholders have a secondary responsibility towards HEPA policies, such as the ministries, the National Institute for Sport Research, and the Physical Education and Sport University.

### Step 2 – Positioning stakeholders in the preliminary schematic model

#### Dutch and Danish cases

In both the Dutch and Danish cases, the local authorities were identified as playing the most central role in the local policy process and were placed centrally in the schematic model (see the grey boxes in Figs. 1 and 2). In both these country cases, the local policymaking process took place at local level, initiated and inspired by the national public health policy. Although this national policy is established by law, the local authorities were in charge of local policymaking, including the HEPA policy, and should therefore take a central position in the schematic model. In the schematic model, the other identified stakeholders were positioned around the local authorities on their respective levels.

#### Romanian case

In Romania, the national level authorities (i.e. the ministries) in the field of sport (to a greater extent), and education and health (to a lesser extent), were identified as being responsible for HEPA policymaking in the case. At local and county level, public administration authorities, together with county representatives of national sport, education and health sectors, and local organisations, were in charge of the implementation of national policies. All the aforementioned stakeholders had some level of (legally binding) accountability in public health promotion.

In local HEPA policymaking, the county and local stakeholders (from both the public and private sectors) of this case played the most important role in embedding the national developed policies, by developing and implementing programs and events. Public-private partnerships are common practice due to the chronic lack of funding in the public system. Therefore (and to increase comparability between country cases), the local (i.e. Mayor and city council) and county (i.e. county council) public administration authorities have been placed in grey boxes (Fig. 3), together with the county representatives of the sport, education and health sectors, while all the other stakeholders are positioned around these central stakeholders.

### Steps 3 – The underlying driving forces

Box 1 provides a description of the nine identified relations, and the underlying driving forces. Three driving forces were distinguished from the nine different identified types of relations existing in the stakeholder network, while developing the HEPA plan. The underlying driving forces were (1) formal relations, (2) informal interaction, and (3) knowledge exchange. Formal relations were hierarchical and formalised by law and the stakeholders in the network needed to act on that. The other two driving forces were more informal. In informal interaction, the focus was especially on communication and the process towards collaboration. The focus of the relations assigned to knowledge exchange was more on research, evaluation of policies, and interventions, which were especially of interest in evidence-informed policymaking.

The identified relations in step three were mapped, resulting in a preliminary final version of the three schematic models of the systems analyses. The focus for each case will be on the driving forces formal relations, informal interaction and knowledge exchange at local level, unless other relations with other levels should be emphasised. The main accountable stakeholder (the public authorities) takes a central place in the systems analysis and therefore are put central in the scheme.

The numbers (X) in the text refer to the numbers in Box 1 and to the numbers of the arrows in the schematic figures, see legend. The final versions of the schematic model of each case are presented in Figs. 1, 2 and 3 (reached after step 5).

#### Box 1 Relations characterised by driving forces

Formal relations

 1) Guidance – giving advice in policy direction and prioritising. Guidance also includes giving advice based on knowledge or strategic planning

 2) Formal acceptance – signing agreements, the hierarchical relations in decision-making

 3) Financial support – hierarchical relations and guidance of allocation of available resources, such as infrastructure

 4) Giving direction by law – guidelines by law and acts and implementation guidelines

Informal interaction

 5) Informal acceptance – creating support between stakeholders, including creating support across sectors about, for example, the agenda

 6) Direct communication – input to policy content, interaction between stakeholders and negotiation across sectors, e.g. wishes or requirements for the policy, and negotiations between stakeholders (e.g. sectors) on issues of concern for the respective stakeholder/sector

 7) Project-based interaction – allocating resources to support the policy plan; includes delivery and support of projects/activities that support the policy or its implementation plan These resource-oriented relations arise by opportunity; the Local Authorities are dependent on the support and activities implemented by other stakeholders to reach specific groups in the community by the policy

Knowledge exchange

 8) Giving feedback – includes evaluation of ideas, advice and priorities given (e.g. qualified input on the policy content and/or on possibilities to fulfil the priorities) and feedback concerning support, commitment, practicalities or former policy implementation

 9) Research utilisation – sharing experience, expertise and scientific evidence, mainly emerging from knowledge institutes; this also includes turning research evidence and evidence from practice into useful information to support the policy

#### Dutch case

Formal relations were mainly characterised by a hierarchical relation and were at the local level, mainly seen between stakeholders within the local authorities and towards other organisations in the whole stakeholder network in the Dutch case (Fig. 1). For example, a guidance (1) arrow was drawn between the city council and the policy officers, because it characterised their dialogue; the city council informs policy officers about political priorities. Important for the policymaking process is also the financial relation (3) between the local authorities and the Regional Sport Service. The latter was directly commissioned to help with the implementation of the HEPA plan.

Informal interaction, especially direct communication (6), was seen in the whole stakeholder network and was especially seen from each of the local organisations towards the local authorities and the regional located services (the identified core stakeholders in the Dutch HEPA policymaking process) and not so much between local organisations. The project-based interaction (7), which covers also implementation of the HEPA policy, occurred mainly between the core stakeholders and schools.

Knowledge exchange was seen between similar stakeholders as the project-based interaction. Research utilisation (9) was mainly taking place between knowledge institutes at regional and national level and towards the sectors in the local authorities. Giving feedback (8), for example on evaluation of previous implemented HEPA plans, was taking place within the local authority.

#### Danish case

In the Danish case, relations and driving forces similar to the Dutch case were extracted. In addition, the explanations of the relations (in terms of driving forces; Box 1) were similar. In the Danish case, the formal relations (hierarchical relations), were mainly seen within the local authorities and from national level stakeholders towards the local authorities (grey box, Fig. 2) and not to other local organisations.

Informal interaction was observed within the local authorities. The directors, together with the Mayor, had a management function and strategic role to prioritise initiatives across the municipality sectors, for which an informal acceptance relation (5) from them to the (executive chiefs of) sectors was identified. This showed once more their accountability as an entity. The local organisations mainly showed relations such as direct communication (6) and project-based interaction (7), i.e. performing activities to support the implementation plan, with the local authorities. As in the Dutch case, the relation ‘project-based interaction’ (8) was essential only in the implementation phase of the policy in the Danish case.

Knowledge exchange was seen within the local authorities, the accountable entity in the development of the HEPA plan, in ‘giving feedback’ (8). At all levels, the research utilisation (9) existed towards the sectors within the local authorities, but not between the knowledge stakeholders (Fig. 2).

#### Romanian case

In Romania, relations and driving forces were extracted, similar to those found in the Netherlands and Denmark. The explanations for financial support (3), informal acceptance (5), direct communication (6) and research utilisation (9) were slightly different in terms of showing a more ad hoc relation, than the more sustained relations in the Netherlands and Denmark.

In the Romanian system, the formal, more hierarchical relations, were mainly observed vertically, from national level stakeholders, representing the sport, education and health sector, towards their county representatives. This was especially the case for the guidance (1) and financial support (3), and is due to the centralised political administrative system in which nationally developed policies are implemented at county and local level. Between the public authorities at county and local level no formal relation, or any of the other identified relations, were identified. However, formal relations were identified from both public authorities towards the local organisations in the form of funding contracts for developing HEPA programs and providing an infrastructure to civil society stakeholders to implement HEPA programs and events, especially those that were ‘Sport for All’ oriented.

The informal interaction relations ran in both directions between national level stakeholders and their county counterparts. For example, county representatives of sport, education and health sectors receive input from their national level counterparts, but also report how the strategic directions outlined from the national level worked in practice, in the field, and what should be adapted, mostly during national strategy meetings or personal contact, not reports. The relations ‘direct communication’ (6) and project-based interaction (7) had both a very broad distribution in the Romanian system, especially between sectors at county level and between local organisations. These interactions had mostly a ‘needs oriented’ and ‘resources-oriented’ character for the implementation of plans and achieving their own organisations’ goals, rather than negotiating on common goals or policy content with respect to HEPA plans.

The knowledge exchange was especially seen at local/county level in the stakeholder network. This implied that the public sector institutions supported the activities of the other sectors, as long as these were in line with their strategy or interests, outlined by the national level strategy. At local level, research utilisation (9) was identified in the process of identification of collaboration potential for reaching goals, and at national level between stakeholders from the sport sector in the development of the national strategy.

### Step 4 – Verification of the models of the systems analyses

In the fourth step, the developed schematic models of the three systems analyses were verified with various key figures and experts.

#### Dutch case

In the Netherlands, in the verification step it was confirmed that all stakeholders and relations were in place and no adaptations were required. This resulted in the schematic model shown in Fig. 1.

#### Danish case

In Denmark, as a result of the verification step in this process the following adaptations were made – (1) some of the project-based oriented relations from local organisations to local authorities and financial relations from national level to local level were verified; (2) the media was added; (3) a simplification of the schematic model was made to promote the dissemination of key relations. All this resulted in the schematic model shown in Fig. 2.

#### Romanian case

In Romania, as a result of the verification step, the following adaptations were made – (1) replacing the County Sport for All Associations from the public to the civil society sector (local organisations), as these institutions are administratively organised as NGOs; (2) addition of one national stakeholder not previously included, i.e. the National Institute for Sport Research; (3) refining the nature of the relations between the stakeholders. This resulted in the schematic model shown in Fig. 3.

### Comparison of the systems analyses of the three cases

Highlights of the main stakeholders and relations are described below with regard to local HEPA policymaking, or when the comparison had implications for the way in which local HEPA policymaking was organised. Tables 3–6 present a comparison between the three country cases for the main stakeholders and the driving forces.

**Table 3:** Comparison of the main stakeholders in local HEPA policymaking between the three country cases

		The Netherlands	Denmark	Romania
HIERARCHICAL POSITIONS IN THE SYSTEMS				
	Authorization and accountability of the local policy	• Local Authority, City council and Board of Mayor and Aldermen	• Local authority, City council, Mayor and political committees	• Accountability not specifically defined locally: National level strategy developed by Ministry Youth and Sport, locally embedded by County Youth and Sport department (main stakeholder in charge of implementing the national developed strategy)
	Final accountability	• Board of Mayor and Aldermen	• Board of Mayor and Aldermen	• No strict accountability
	Allocation of tasks	• Civil servants in sectors • Municipality services • Local organisations	• Civil servants in sectors • Municipality services • Local organisations	• Local organisations
STAKEHOLDERS WITH SIMILAR POSITIONS IN THE SYSTEMS				
Local/ County	Local & County authority	• City council • Board of Mayor and Aldermen • Sectors at local level • Municipality services	• City council • Political committees • Mayor and Directors • Sectors at local level • Municipality services	• Mayor and City Council • County Council • Sectors at county level, representative of sectors national level
	Part of municipality services	• Combination functions • Centres for youth and family	• Schools • Day care • Health service • Social neighbourhood co-workers	
	Local organizations, including private and civil society	• Schools and day care • Sport clubs • Health and Care organisations • Private partners • Local councils and citizen’s platform	• Local councils, such as sports, youth • Interest groups, e.g. local sports associations • Private partners • Media	• Sport equipment companies • Running clubs • Cycling clubs • Paralympics sport organisation • Media • Student NGOs
Regional	Regional stakeholders influencing local level	• Public Health Service • Regional Sport Services	• Research Centre for Prevention and Health	• County Sport for All Association
National	National stakeholders influencing local level	• Ministry of Health, Welfare and Sports • Health Inspectorate • Knowledge institutes: National Institute for Public Health and the Environment, National Institute for Physical Activity and Sports^a^, National Youth on Healthy Weight Bureau	• Ministry of Health • Danish Nature Agency • Healthy cities network • Local Government Denmark • Universities • Danish Health and Medicines Authorities	• Ministry of Youth and Sport • Romanian Sport for all Federation • Ministry of Education • Ministry of Health • National Institute for Sport Research • National Physical Education and Sport University • Private Sector and Civil Society

^a^Since January 2016: Knowledge Centre for sport Netherlands

#### Main stakeholders

In the schematic models of each of the country cases, the main stakeholders are indicated in bold (Figs. 1, 2 and 3). At local/county level, the three cases showed similarities and differences in the structure of the system and the stakeholders, with mostly similarities between the Dutch and the Danish cases, and mostly differences compared with the Romanian case (especially within the public authorities). Although the different sectors in Romania were identified at county level, their position was similarly related to the sectors identified at the local level in Denmark and the Netherlands. In the Netherlands and Denmark, within the public authorities, two other stakeholders were identified (besides the council and sectors), i.e. the Board of the Mayor and Aldermen/political committees and municipality services. The local authorities were identified as the entity with the decision-making power over the entire local policymaking process, established by law and, therefore, being accountable. As a municipal entity, they were expected to take the initiative to start developing local implementation policies. However, the way in which the actual work was executed was left to the municipality at stake. For example, in the Netherlands, the municipality could assign the development of the HEPA implementation plan to another stakeholder, such as the Regional Sport Service. This was not the case in the Danish system, where the healthcare sector was accountable for the coordination of the development of the implementation plan. In Romania, a very different picture emerged. All local and county level stakeholders had some level of accountability in the implementation of public health policies, based on the nationally proposed policy strategy.

In all three country cases, schools showed to have an important role in HEPA policymaking. However, how schools are embedded in the policy network differed between the three countries, which implies a different role and influence of schools. In Denmark, schools were part of the public authorities, whereas, in the Netherlands, schools were identified as separate stakeholders at local level. Schools were also identified in Romania; however, in this system, the County School Inspectorate (an institution directly subordinate to the Ministry of Education) represented them. Other local organisations were holding a similar position in the three systems.

Knowledge stakeholders were identified at all levels in all three countries, but how they were positioned differed. This might affect knowledge exchange accordingly.

National level stakeholders appeared to have most influence on the local HEPA policymaking process in Romania. Although this country had two administrative authorities at local level (the local and county public authorities), none of these authorities had decision-making power similar to that of the Dutch and Danish local authorities; this is due to a lack of structure within the Romanian organisations to make these decisions. Also, they were not accountable by law for the HEPA policy process; in Romania, national level stakeholders were in charge of the policy plan, developing strategies to be implemented at local/county level. Furthermore, in Romania, county level organisations in the field of Sport, Education and Health, appeared to have the most influence (mandated by the nationally developed strategies), whereas in the Dutch and Danish cases, mainly local level stakeholders took part in the HEPA policy process, which again implies major differences in the local HEPA policy process between these countries.

Whereas in the Romanian case the HEPA policy plan was based on a national strategic sport plan, this plan was based in the Netherlands and Denmark on the local public health plan. This implies a difference in how HEPA policies were organised and embedded. The identified type of stakeholders involved in local HEPA policymaking in the three cases support this implication. In the Netherlands and Denmark, stakeholders mainly focused on (public) health, whereas in Romania, they mainly focused on sports and (to some extent) physical activity. Also, in the Dutch and Danish systems, specific sport stakeholders were identified in the policy process, even though the positions and relations of these stakeholders in the systems differed. For more information on a comparison between main stakeholders, see Table 3.

#### Relations between stakeholders in local HEPA policymaking

Three driving forces were distinguished, representing the nine identified relations. The driving forces identified in the systems were similar for the three cases: formal relations, informal interaction, and knowledge exchange. Some differences emerged in the explanation of the relations in the Romanian case, which were mainly due to the more temporary project-based nature of the relations. Therefore, the Romanian case showed a less structural character of the relations compared with the other country cases, which is not directly visible in the figures. Nevertheless, these differences tended to affect the entire system in Romania; for example, implementation of the local HEPA policies due to differences in the national and local administrative structures and the roles assigned by law to the national, county/regional and public institutions with regard to the responsibilities they have in HEPA promotion.

The comparison between relations is based on the three driving forces and focuses on the interaction between the stakeholders and their implications for collaboration and knowledge exchange towards evidence-informed policymaking in each of the three country cases.

#### Formal relations

Taking a closer look at the differences, two main differences appeared. First of all, the influence of national level, which seemed to be higher in Romania and Denmark. In the Netherlands, the local level appeared to be a more separate entity. In addition, in Romania, a hierarchical differentiation seemed to exist between national and county level sectors and not between the public authority at local and county level.

Second, the local organisations in Romania seemed to have most influence on the actual implementation of plans at the local level, because of the formalised acceptance of the plans towards the local organisations in Romania. Table 4 presents a comparison between the identified hierarchical relations in local HEPA policymaking in the three countries – the influence of the different identified levels are shown.

**Table 4:** Formal relations in local HEPA policymaking across the three country cases

DRIVING FORCE: FORMAL RELATIONS				
		The Netherlands	Denmark	Romania
Within local level	Guidance (1)	• Board of Mayor and Aldermen > Sectors • Sectors > Municipality services • City council > Sectors	• Political Committees > Sectors • Sectors > mayor • Sectors > Municipality Services	
	Formal acceptance (2)	• City Council > Board of Mayor and Aldermen	• City Council > Political Committees	• Local authorities > Local organisations
	Financial support (3)	• Local Authority > Regional Public Health Service & Regional Sport Service	• Patient organisations > sectors	• Local authorities > Local organisations
From regional towards local level	Formal acceptance (2)			• County authorities > Local organisations
	Financial support (3)	• Other municipalities > Regional Public Health Service & Regional Sport Service		• County authorities > Local organisations
From national towards other levels	Guidance (1)		• Danish Health and Medicines Authority > Local Authority • Local Government Denmark > Sectors • Knowledge institutes > Sectors • Healthy Cities Network > Sectors	• Romanian Government > Ministries • Ministry of Education > County school inspectorate • Ministry of Health > County public health • Ministry of Youth and Sports > County Youth and Sport & Romanian Sport for All Federation & National Institute for Sport Research • Romanian Sport for All Federation > County Sport for All Association • Private sector and civil society > Local organisations • Physical Education and Sport University > Ministry of Youth and Sport
	Formal acceptance (2)	• Health inspectorate > Regional Public Health Service • Health inspectorate > Local authorities	• Danish Nature Agency > Sectors	• Private Sector and Civil Society > Local organisations
	Financial support (3)	• Ministries > Municipalities	• Ministries > Municipalities • Danish Nature Agency > Sectors • NGOs > Sectors	• Ministry of Education > County Sector Education • Ministry of Health > County Sector Health • Ministry of Youth and Sports > County Sector Sport & Romanian Sport for All Federation • Romanian Sport for All Federation > County Sport for All Association
	Giving direction (4)	• Ministries > Local authorities • National Institute for Public Health and the Environment > Regional Public Health Service	• Ministries > Local authorities	• Ministry of Education > County Sector Education • Ministry of Health > County Sector Health • Ministry of Youth and Sports > County Sector Sport • Romanian Sport for All Federation > County Sport for All Association

#### Informal interaction

In all three countries, much informal interaction existed between the different stakeholders, although the strengths and intensity of the relations were not revealed in this study. In the Netherlands and Denmark, the communication relations were mainly identified at local level and (to some extent) between local and regional level in the Netherlands; again, this implies the self-regulated entity at local level. In Romania, these relations were seen across all three levels, implying a different influence of the national level stakeholders on the local level stakeholders. In the Netherlands and Denmark, the sectors within the public authorities and the regional services in the Netherlands seemed to be the central stakeholder for project-based interaction, whereas in Romania, much of the implementation was initiated by the local civil society organisations, depending on the allocation of resources from the public sector, and some resources from private companies. Table 5 presents a comparison of the identified relations, based on informal communication, in local HEPA policymaking between the three country cases, showing the more informal relations among stakeholders.

**Table 5:** Informal interaction in local HEPA policymaking across the three country cases

DRIVING FORCE: INFORMAL INTERACTION				
		The Netherlands	Denmark	Romania
Within local level	Informal acceptance (5)	• Sectors > City council • Regional public health service > Care and welfare organisations	• Mayor > Executive chiefs sectors • Directors > Executive chiefs sectors • Municipality services > Sectors	• Local authorities > Local organisations
	Direct communication (6)	• Between Board of Mayor and Aldermen • Between sectors • City council <-> sectors • Local council <-> sectors • Local council <-> city council • Private partners <-> Board of Mayor and Aldermen • Private partners <-> Sectors • Private partners <-> Regional public health service	• Between political committees • Between sectors • Directors <-> Mayor • Interest groups <-> City council • Patients organisations <-> City council • Patient organisations <-> Sectors • Local councils <-> Political committee • Local councils > Sectors	• Between local organisations • Local organisations <-> City council, County council, Sectors education and health • Between local organisations
	Project-based interaction (7)	• Schools <-> Sectors	• Interest groups <-> Sectors • Center of volunteers <-> Sectors • Private parties <-> Sectors	• Local authorities <-> Local organisations • Between local organisations
Between local and regional level	Informal acceptance (5)			• County authorities > Local organisations
	Direct communication (6)	• Within regional public health service • Regional sport service <-> Local authorities • Regional public health service <-> Regional sport service		• County council <-> County sector sport • Local organisations > County Council & Children Palace & Sector Health • County Sport for All Association <-> Local organisations
	Project-based interaction (7)	• Regional public health service <-> Sectors • Regional sport service <-> Sectors		• Between county sectors *•* County authorities <-> Local organisations *•* County Sport for All Association <-> Local organisations
From national towards other levels	Direct communication (6)	• National Institute for Physical Activity and Sport^a^ <-> Sectors • Ministry of Health, Welfare and Sports <-> National Institute for Public Health and the Environment, National Institute for Physical Activity and Sports^a^, Youth on Health Weight, Health inspectorate, other ministries	• Danish Health and Medicines Authority <-> Local authorities • Danish Nature Agency <-> Sectors • Ministry of Health <-> Local Government Denmark • Ministry of Health <-> Danish Health and Medicines Authority	• Romanian Sport for All Federation <-> County Sport for All Association • Private sector and civil society <-> Local organisations
From national towards other levels	Project-based interaction (7)	• National Institute for Physical Activity and Sport^a^ <-> Sectors		

^a^Since January 2016: Knowledge Centre for sport Netherlands

#### Knowledge exchange

The relation research utilisation was identified in all three countries in the implementation phase of the HEPA policy in the way of delivering support. The relations emerged between several stakeholders in all three country cases.

The way research utilisation was distributed differed between the countries and the core stakeholders seemed to be more related in this regard in the Netherlands and Denmark. How this relation was embedded in the systems might indicate a different support system of the development and implementation of local HEPA policies and might be dependent on the core stakeholders for HEPA policymaking in each of the country cases. A comparison of the identified resources relations in local HEPA policymaking between the three countries is presented in Table 6.

**Table 6:** Knowledge exchange in local HEPA policymaking across the three country cases

DRIVING FORCES: KNOWLEDGE EXCHANGE				
		The Netherlands	Denmark	Romania
Within local level	Giving feedback (8)	• Municipality services > Sectors • Sectors > Board of Mayor and Aldermen • Board of Mayor and Aldermen > City council	• Municipality services > Sectors • Sectors > Political committees • Political committees > City council	
	Research utilisation (9)		• Local authorities <-> Media	• Local authorities <->Local organisations
	Research utilisation (9)	• Regional Public Health Service <-> Sectors • Regional Sport Service <-> Sectors • Regional Sport Service <-> Regional Public Health Service	• Research Centre for Prevention and Health > Local authorities	• County authorities <-> Local organisations
From and between national and other levels	Giving feedback (8)			• Ministry of Education <-> County Sector Education • Ministry of Health <-> County Sector Health • Ministry of Youth and Sports <-> County Sector Sport • Romanian Sport for All Federation <-> County Sport for All Association • Physical Education and Sport University > Ministry of Youth and Sport Private Sector and Civil Society <-> Local organisations
	Research utilisation (9)	• National Institute for Physical Activity and Sport^a^ <-> Regional Public Health Service • Universities <-> Regional Public Health Service	• Local Government Denmark <-> Sectors • Universities <-> Sectors • Healthy Cities Network <-> Sectors	• Romanian Sport for All Federation <-> County Sport for All Association

^a^Since January 2016: Knowledge Centre for sport Netherlands

## Discussion

The main findings of this study are two-fold. Firstly, it increases the understanding of systems in local HEPA policymaking in different countries, in terms of involved stakeholders, their relative positions, and the types of relations between them. Second, it shows differences and similarities between the three country cases. Earlier studies have shown which groups of stakeholders form part of local policymaking and (to some extent) the complexity of the local policy process [11, 15, 29, 30, 32]. Our analysis further elucidates the positions of and relations between stakeholders in the policy network of local HEPA policymaking, placing the policy network in comparable schematic models.

This analysis provides a starting point in the discussion of the stakeholder network with the involved stakeholders with regards to HEPA policymaking. The schematic models highlight the explicit knowledge-exchange relations in the stakeholder network, which are considered important in the interactive model for the uptake of evidence [20]. The analysis shows where interaction and collaboration already exists (or was lacking) between the involved stakeholders and, hence, where this can be stimulated to increase the uptake of evidence [8, 43–45]. In addition, the schematic models provide information on accountability in the stakeholder network, the formal relations, providing information on how to influence knowledge exchange from that perspective [8]. Whereas in Denmark and the Netherlands local HEPA policymaking took place at the local level and the local authority was held accountable for the process by law, in Romania, the strategy was proposed at the national level, albeit implemented at the local level, mainly by local organisations.

Comparing country cases in this specific way also sheds new light on structural differences in local HEPA policy networks and the policymaking processes between these European countries. In the Netherlands and Denmark, there seems to be a good foundation for knowledge exchange because of the structural basis of the stakeholder network and the different relations (formal, informal and knowledge exchange) between the knowledge institutes and the accountable entity in the HEPA policymaking process. In Romania, the implementation of the HEPA policy was more project based, introduced by local organisations on an ad hoc basis and dependent on national strategic decisions and allocation of resources. This implies major differences in the support systems of implementing local HEPA policies, being a more structural and locally embedded process in the Netherlands and Denmark compared to that in Romania. Furthermore, in Romania, many more stakeholders from the sports sector were identified compared with health stakeholders in the Netherlands and Denmark, implying a different focus in HEPA policymaking. The lower variety in sectors together with the more ad hoc basis in Romania also implies a less integrated cross-sectoral approach as is advocated for the development of an effective public health policy [3–5].

In addition, relations and interactions between stakeholders in the stakeholder network are highly relevant when an increase in collaboration, and thus in knowledge exchange, is desired [9]. One of the essential relations for the uptake of (research) evidence is knowledge exchange. However, relations specifically focusing on knowledge exchange were only one of the nine types of identified relations and mainly existed between national/regional towards local stakeholders. This implies that most existing relations between stakeholders do not explicitly focus on knowledge exchange. However, these other relations can offer good opportunities for day-to-day knowledge exchange in the real life context. As indicated in the reviews by Oliver et al. [10] and Innvaer et al. [46], interaction and relations within the stakeholder network are seen as the main facilitators for evidence-informed policymaking. This was also found in a recent study based on the first phase of the REPOPA project [47]. Hence, systems analyses can be seen as an instrument to reveal opportunities for improving knowledge exchange at the local level.

Although relations on knowledge exchange and communication between stakeholders in the system were identified, we did not collect information on the strength of the relations between stakeholders as would be identified by a stakeholder network analysis [28, 31, 48]. This may mean that, even though an organisation may belong to the stakeholder network, it is possible that an individual belonging to that organisation has no structural relations in the specific local stakeholder network. In other words, the strength of relations between (individuals within) organisations might differ and, in turn, so will the influence of a stakeholder in the overall policy process. In this study, however, we focused on unravelling the relations between stakeholder organisations in a local stakeholder network and not on strength of relations between individuals. The simplified representation of reality (arising from the methodology, in combination with the aim of this study), can be seen as a strength, because this approach helped to better identify differences and similarities between the countries in local HEPA policymaking.

A possible limitation of this study is the particularity of the case selected in each country. Each of the countries chose the most suitable local HEPA policy in their country, taking into account the inclusion criteria. However, complete similarity of real-life cases is not feasible. Some of the differences found were challenging. In the Netherlands and Denmark, the focus of the HEPA policy was on public health, including physical activity. The focus in the Romanian case was on sports and ‘Sport for All’ (including HEPA) and was organised as a responsibility of the sport sector.

To generate a broader generic picture of the country policymaking system and to underpin the selection of the cases, policy documents of other municipalities were also analysed. For example, in Romania, the local (Mayor and city council) and county authority (county council, and county representatives of national level sectors) are organised in a way similar to that in the Netherlands and Denmark. Also, in Denmark and the Netherlands, the outline of the schematic model of the systems analysis is similar across municipalities, even though the details differ. This contributes to the generalisability of our findings. Therefore, it is expected that the overall outline of the schematic models will be similar across municipalities in these three countries.

The developed four-step guideline for systems analysis and the identified relations might be a valuable starting point for analysing other cases, both for the countries presented here as well as for other European countries who would like to increase insight into local HEPA policymaking, or other policy areas. A systems analysis, carried out by applying the four-step guideline, might be a promising instrument to initiate and enhance the communication and collaboration between stakeholders. The schematic model that results from it, represents the complex problem in the policymaking process. The information from the schematic models of the systems analyses provide baseline information on the network’s systems characteristics, organisational network, relations, communication, collaboration and knowledge exchange. This information can be valuable for the stakeholders involved in local HEPA policymaking to understand how to approach and interact with other stakeholders in the policy process [12, 49]. This might help to overcome the gap between research and policy communities [11, 21, 50, 51], and to increase the impact of evidence in the policy process [52–54].

Furthermore, the systems analysis brings added value to understanding local HEPA policymaking in different European countries and creates an opportunity for successful intervention development [9]. The similarities between countries provide important information to build an intervention to stimulate collaboration among stakeholders; a policy game intervention can be such an intervention. Studies have shown that games might positively influence collaboration, and the understanding of the relations and dynamics between stakeholders, and allow to experiment in a safe environment [55]. The highlighted differences between countries are useful to apply a policy game aiming at collaboration and knowledge exchange. A systems analysis is a first step in providing input for the development of a policy game intervention [55].

## Conclusions

The three systems analyses and their representation in the schematic models provide a general picture of the functioning of stakeholder networks in local HEPA policymaking in three European country cases. The systems analyses enhance our understanding of how local stakeholder networks function. The analysis increases insight into the structure and processes of local HEPA policymaking networks by offering a simplified version of the complex process and the relations that exist between stakeholders involved; this also helps to compare the different systems. The results of our study can contribute to establishing, maintaining or even improving evidence-informed health policies. These insights can also be used to develop interventions that may facilitate the interaction and collaboration between stakeholders in the local HEPA network and, thereby, help enhance knowledge exchange and uptake of evidence to develop more effective public health policies.

## Abbreviations

HEPA: health-enhancing physical activity
REPOPA: REsearch into POlicy to enhance Physical Activity

## References

[1] Dahlgren G, Whitehead M (1991). Policies and Strategies to Promote Social Equity in Health.

[2] Kickbusch I, Buckett K (2010). Implementing Health in All Policies.

[3] Kickbusch I, McCann W, Sherbon T (2008). Adelaide revisited: from healthy public policy to Health in All Policies. Health Promot Int.

[4] Storm I, Verweij A, Van der Lucht F (2011). Integraal gezondheidsbeleid op lokaal niveau. Wat weten we en hoe nu verder?. RIVM Briefrapport 270161004.

[5] Kohl HW (2012). The pandemic of physical inactivity: global action for public health. Lancet.

[6] Lavis JN (2008). Evidence-informed health policy 1 - synthesis of findings from a multi-method study of organizations that support the use of research evidence. Implement Sci.

[7] Lomas J (2000). Using 'linkage and exchange' to move research into policy at a Canadian foundation. Health Aff.

[8] Bryson JM (2004). What to do when Stakeholders matter. Public Manage Rev.

[9] Hyder A (2010). Stakeholder analysis for health research: case studies from low- and middle-income countries. Public Health.

[10] Oliver K (2014). A systematic review of barriers to and facilitators of the use of evidence by policymakers. BMC Health Serv Res.

[11] de Goede J (2012). Looking for interaction: quantitative measurement of research utilization by Dutch local health officials. Health Res Policy Syst.

[12] Jansen MW (2010). Public health: disconnections between policy, practice and research. Health Res Policy Syst.

[13] Shearer JC, Dion M, Lavis JN (2014). Exchanging and using research evidence in health policy networks: a statistical network analysis. Implement Sci.

[14] Bowen S, Zwi AB (2005). Pathways to "evidence-informed" policy and practice: a framework for action. PLoS Med.

[15] Jansen MW (2008). Collaboration between practice, policy and research in local public health in the Netherlands. Health Policy.

[16] Walter I, Davies H, Nutley S (2003). Increasing research impact through partnerships: evidence from outside health care. J Health Serv Res Policy.

[17] Kothari A, Armstrong R (2011). Community-based knowledge translation: unexplored opportunities. Implement Sci.

[18] Contandriopoulos D (2010). Knowledge exchange processes in organizations and policy arenas: A narrative systematic review of the literature. Milbank Q.

[19] de Goede J (2010). Knowledge in process? Exploring barriers between epidemiological research and local health policy development. Health Res Policy Syst.

[20] Hanney SR (2003). The utilisation of health research in policy-making: concepts, examples and methods of assessment. Health Res Policy Syst.

[21] de Leeuw E (2008). Theoretical reflections on the nexus between research, policy and practice. Crit Public Health.

[22] Weiss CH (1979). The many meanings of research utilization. Public Adm Rev.

[23] Davies HTO, Nutley SM, Smith PC (2010). What Works? Evidence-Based Policy and Practice in Public Services.

[24] Daugbjerg SB (2009). Promotion of physical activity in the European region: content analysis of 27 national policy documents. J Phys Act Health.

[25] Bull FC (2014). National policy on physical activity: the development of a policy audit tool. J Phys Act Health.

[26] Edwards P, Tsouros A (2006). Promoting Physical Activity and Active Living in Urban Environments.

[27] Hakamäki P (2015). Promotion of Physical Activity in Municipalities 2010-2014.

[28] Hoeijmakers M (2007). Local health policy development processes in the Netherlands: an expanded toolbox for health promotion. Health Promot Int.

[29] Edelenbos J (2005). Managing stakeholder involvement in decision making: a comparative analysis of six interactive processes in the Netherlands. J Public Adm Res Theory.

[30] Oliver K (2013). Who runs public health? A mixed-methods study combining qualitative and network analyses. J Public Health (Oxf).

[31] Kok G (2015). Influencing organizations to promote health applying stakeholder theory. Health Educ Behav.

[32] Eklund Karlsson L, et al. Involvement of external stakeholders in local health policymaking process: a case study from Odense Municipality, Denmark. Evid Policy. 2016. https://doi.org/10.1332/174426416X14609162710134.

[33] Friedman AL, Miles S (2002). Developing Stakeholder Theory. J Manag Stud.

[34] Bryson JM, Crosby BC, Middleton SM (2006). The design and implementation of cross-sector collaborations: propositions from the literature. Public Adm Rev.

[35] Duke RD, Geurts JLA (2004). Policy Games for Strategic Management Pathways into the Unknown.

[36] Peters V, van de Westelaken M (2011). Spelsimulatie - Een beknopte inleiding in het ontwerpproces.

[37] Stoppelenburg A, de Caluwé L, Geurts JLA (2012). Gaming Organisatieverandering met spelsimulaties.

[38] Geurts JLA, Duke RD, Vermeulen PAM (2007). Policy Gaming for Strategy and Change. Long Range Plann.

[39] Aro AR (2016). Integrating research evidence and physical activity policy making—REPOPA project. Health Promot Int.

[40] Goodman LA (1961). Snowball sampling. Ann Math Stat.

[41] Adami MF, Kiger A (2005). The use of triangulation for completeness purposes. Nurse Res.

[42] Denzin NK (1989). The Research Act: A Theoretical Introduction to Sociological Methods.

[43] Exworthy M (2008). Policy to tackle the social determinants of health: using conceptual models to understand the policy process. Health Policy Plan.

[44] Lundin M, Öberg P (2013). Expert knowledge use and deliberation in local policy making. Policy Sci.

[45] Bull FC (2004). Developments in National Physical Activity Policy: an international review and recommendations towards better practice. J Sci Med Sport.

[46] Innvaer S (2002). Health policymakers’ perceptions of their use of evidence: a systematic review. J Health Serv Res Policy.

[47] van de Goor LAM, et al. Barriers and facilitators in evidence use in public health policy. Results from a study across six EU countries. Health Policy. Accepted Jan 2017. 10.1016/j.healthpol.2017.01.003 PMC5754321 28139253

[48] Brugha R, Varvasovszky Z (2000). Stakeholder analysis: a review. Health Policy Plan.

[49] Varvasovszky Z, Brugha R (2000). How to do (or not to do)… A stakeholder analysis. Health Policy Plann.

[50] Cooke J (2015). On-going collaborative priority-setting for research activity: a method of capacity building to reduce the research-practice translational gap. Health Res Policy Syst.

[51] Morestin F (2015). Knowledge Sharing and Public Policies: A Representation of Influence Processes.

[52] Brownson RC (2006). Researchers and policymakers: travelers in parallel universes. Am J Prev Med.

[53] Liverani M, Hawkins B, Parkhurst JO (2013). Political and institutional influences on the use of evidence in public health policy. A systematic review. PLoS One.

[54] Orton L (2011). The use of research evidence in public health decision making processes: systematic review. PLoS One.

[55] Geurts JLA, Joldersma C (2001). Methodology for participatory policy analysis. Eur J Oper Res.

[56] Edwards N (2012). Challenges of ethical clearance in international health policy and social sciences research: experiences and recommendations from a multi-country research programme. Public Health Rev.

[57] Aarts MJ (2011). Children, physical activity and the environment: Opportunities for multi-sector policy.

[58] Hoeijmakers M (2005). Local Health Policy Development Processes: Health Promotion and Network Perspectives on Local Health Policy-making in the Netherlands.

[59] van Egmond S (2010). Science and Policy in Interaction: On Practices of Science Policy Interactions for Policy-making in Health Care.

[60] Fischer-Nielsen B. Kommunalpolitik. Copenhagen: Columbus; 2005.

[61] Lundtorp S, Rasmussen M (2007). Rigtigt kommunalt – ledelse I kommuner og amter fra reform til reform.

